# Fibrosis Severity and Pro-fibrotic Gene Expression in Uterine Leiomyomas: Relationship with Self-reported Skin Color/Race and Genomic Ancestry

**DOI:** 10.1007/s43032-025-02034-y

**Published:** 2026-02-02

**Authors:** Ana Claudia M. Scalioni, Luciana Bastos-Rodrigues, Elaine Sousa, Tays F. Guedes, André L. Caldeira-Brant, Luiz De Marco, Wiviane A. Assis, Fernando M. Reis

**Affiliations:** 1 Department of Obstetrics and Gynecology, Division of Human Reproduction, Universidade Federal de Minas Gerais, Av. Alfredo Balena 110–9° andar, Belo Horizonte, MG 310130-100 Brazil; 2 Department of Nutrition, Universidade Federal de Minas Gerais, Belo Horizonte, Brazil; 3 Department of Surgery, Universidade Federal de Minas Gerais, Belo Horizonte, Brazil

**Keywords:** Leiomyoma, Skin color, Race, Genomic ancestry, Fibrosis, Collagen, Fibronectin, Versican

## Abstract

Uterine leiomyomas (ULM) are highly prevalent benign gynecologic tumors and the leading indication for hysterectomy. Black women experience a disproportionate impact of ULMs compared to White women, but the mechanisms underlying this racial disparity remain elusive. The aim of this study was to evaluate the degree of fibrosis and the expression of pro-fibrotic genes in ULMs from Brazilian women according to self-reported color/race and genomic ancestry. Study participants (*n* = 30) were divided into three groups (*n* = 10) according to self-declared skin color/race: Black, Brown, or White. ULM tissue was collected through hysterectomy. Fibrosis was quantified by Masson's trichrome staining and the expression of pro-fibrotic genes *COL1A1* (type 1 collagen), *FN1* (fibronectin 1) and *VCAN* (versican) was assessed by qPCR. The genetic ancestry was determined by typing DNA for a panel of 40 validated ancestry-informative biallelic short insertion/ deletion DNA polymorphisms. The three groups presented homogeneous clinical and demographic data. The proportion of fibrotic tissue in the ULM histological sections was similar in the three groups. The mRNA levels of type 1 collagen were lower in Black than in Brown group (*p* < 0.05) while fibronectin and versican expression did not change according to self-reported color. There was no correlation between fibrosis or pro-fibrotic gene expression and the proportion of European, African or Amerindian genomic ancestry of the participants. These findings suggest that differences in the amount of fibrosis or the expression of pro-fibrotic genes by the time of hysterectomy are unlikely to explain racial disparities in the burden of uterine leiomyomas among Brazilian women.

## Introduction

Uterine leiomyomas (ULMs), also termed fibroids, are highly prevalent benign gynecologic tumors, affecting about 70–80% of women during their lifetime [1]. It is estimated that 20–50% of all women with ULMs experience related symptoms, which can seriously affect their quality of life [2]. The main symptoms associated with ULMs are abnormal uterine bleeding, chronic pelvic pain, infertility and obstetrical complications [3, 4]. ULMs are the leading indication for hysterectomy, but despite its effectiveness the loss of reproductive potential and a significant morbidity are major limitations of this intervention [5].

ULMs originate from the smooth muscle layer of the uterus and probably develop from a single transformed myometrial smooth muscle cell [6, 7]. They are characterized by abundant extracellular matrix (ECM) accumulation [8], and alterations in ECM composition significantly contribute to tumor growth [9, 10]. Tumor bulk results from a disorder of fibrosis [11–14] with the production of large amounts of ECM proteins such as collagens, fibronectin and proteoglycans [15]. Of particular importance, collagen type 1/subtype 1A1 (*COL1A1*), fibronectin 1 (*FN1*) and the proteoglycan versican (*VCAN*) are overexpressed by ULM cells and have been implicated in the pathophysiology of ULMs not only as components of the ECM but also promoters of cell proliferation and fibrosis [15–17].

African ancestry is considered a key risk factor for the development of ULMs. The cumulative incidence of ULMs for Black women exceeds that for White women by 20% in their 30 s and by 10% in their late 40 s [1]. In addition, African American women have ULMs diagnosed at earlier ages and are more likely to be symptomatic and non-responsive to medical treatments than White women [18]. The size and growth rates of ULMs are greater in African American women, who undergo surgical intervention more often than other racial groups [19].

Despite the growing knowledge of the pathophysiology of ULMs, the underlying mechanisms of racial disparities in their incidence, progression and clinical manifestations are largely unknown [20]. A study in the United States revealed novel protein traits like tetratricopeptide repeat protein 38 (TTC38) in Black patients with ULMs and found evidence that *MED12* pathogenic variants, which are more prevalent in the Black population and predispose to ULM development, also correlate with increased tissue fibrosis [21]. Clearly, more information is needed on diverse populations, including those with a large proportion of mixed-race individuals. Thus, the aim of this study was to evaluate the degree of fibrosis and the expression of pro-fibrotic genes *COL1A1*, *FN1* and *VCAN* in leiomyomas from Brazilian women according to both self-reported skin color/race and genomic ancestry.

## Materials and Methods

### Study Design and Participants

This prospective cross-sectional study included 30 women with indication of hysterectomy for uterine myomatosis. The patients were recruited at the Gynecology Units of two general hospitals in Belo Horizonte, Brazil, from November 2022 to April 2023. Women with malignant neoplasm, current use of corticosteroids, current pituitary blockade with GnRH analogue or postmenopausal were excluded.

The participants were divided according to self-reported skin color/race into Black, Brown or White. The color assignation was obtained in answer to the closed question “What is your color/race?”, as done in the Brazilian population census [22]. The clinical characteristics of the study participants are shown in Table 1.

**Table 1:** Clinical characteristics of the study participants

	Black *n* = 10	Brown *n* = 10	White *n* = 10	*P* value
Age (years)	42.2 ± 4.9	45.1 ± 3.5	44.8 ± 4.2	0.323
Menarche (years)	12.3 ± 1.9	13.4 ± 1.6	12.3 ± 2.6	0.432
Weight (kg)	77.3 ± 17.8	74.3 ± 15.2	79.5 ± 12.8	0.609
Body mass index (kg/m^2^)	28.6 ± 6.3	28.0 ± 5.2	30.2 ± 4.8	0.442
Uterine volume (cm^3^)	394 ± 296	394 ± 272	333 ± 203	0.856
Hemoglobin level (g/dL)	13.1 ± 2.4	11.9 ± 2.5	13.5 ± 0.8	0.318
Menstrual cycle (days)	7 (6—8)	7 (6—7)	7 (7—8)	0.667
Dysmenorrhea (VAS)	8.0 (4.5–9.0)	9.0 (6.5–10.0)	8.0 (0.0–10.0)	0.765
Pelvic pain (VAS)	6.0 (0.0—8.5)	6.0 (0.0–10.0)	5.0 (0.0—8.3)	0.758
Parity	2 (2—3)	2 (1—4)	3 (2—3)	0.663
Systolic blood pressure (mmHg)	127 (117—148)	120 (117—132)	122 (117—138)	0.762
Diastolic blood pressure (mmHg)	78 (75—81)	80 (73—82)	80 (70—87)	0.940

Values are given as mean ± SD or median (interquartile range)

*VAS* Visual Analogue Scale

### Sample Collection

One core tissue sample of the largest ULM type 2 to 6 [23] was obtained immediately after removal of the uterus. The collected tissue fragments were split into two portions that were stored in fixative solution (Histochoice®, Sigma) and RNA preservation solution (RNAlater®, Sigma), respectively.

Blood samples (1–2 ml) were collected from a peripheral vein in EDTA-coated tubes and stored at −20 °C for up to 30 days until DNA extraction.

### DNA Extraction and Ancestry Genotyping

Genomic DNA was extracted from peripheral blood samples using standard protocols. The quality and quantity of each DNA sample were tested by NanoDrop ND −2000 UV–Vis Spectrophotometer (Thermofisher, Waltham, MA) [24].

All individuals had their genetic ancestry determined by typing DNA with a panel of 40 validated ancestry-informative biallelic short insertion/deletion DNA polymorphisms (InDels) [25]. Amplicons were sized using an ABI3130 DNA Sequencer (Applied Biosystems) and analyzed using the GeneMapper Software version 3.7. As a population clustering algorithm, we used the Structure program version 2.3, available at < http://pritch.bsd.uchicago.edu/structure.html > to infer the structure of each population and allocate individuals to the three different ethnic groups: Africans, Europeans and Amerindians [22, 26].

### RNA Extraction, Complementary DNA Synthesis, and Semi-quantitative PCR

Total RNA was isolated using the TRIzol® protocol and quantified by light absorbance at 260 nm (NanoDrop—Thermo Fisher Scientific, Wilmington, Delaware, USA). One μg of RNA was pretreated with DNase I, Amplification Grade for 15 min (CAT: 18068015; Invitrogen, Carlsbad, CA, USA) to remove undesired genomic DNA contamination. First-strand complementary DNA (cDNA) was synthesized from 750 ng of DNase I-treated total RNA using Superscript IV first-strand synthesis system (CAT: 18091050; Invitrogen, Carlsbad, CA, USA).

Real-time PCR was carried out in an ABI-Prism 7500 Sequence Detection System using the fluorescent dye Power SYBR Green Master Mix Kit (Invitrogen Life Technologies, Carlsbad, CA, USA). The PCR parameters were: [stage 1] a cycle of 95 °C/10 min; [stage 2] 40 cycles of 95 °C/15 s, 60 °C/15 s and 72 °C/20 s; [Stage 3] 95 °C/15 s, 54 °C/15 s and 95 °C/15 s. The gene encoding the ribosomal protein S26 was used as the internal control.

Table 2 shows primer sequences used for PCR amplification. Primers were designed to span two sequential exons and thus anneal only to cDNA. The specificity of PCR products was confirmed by single peak dissociation curves. Threshold cycle (Ct) values were normalized to S26 (ΔCt), and each sample value was reported as 1/ΔCt.

**Table 2:** Primer sequences used for PCR amplification

Gene	Forward primer	Reverse primer	Length (bp)
*S26*	TGTGCTTCCCAAGCTGTATGTGAA	CGATTCCTGACTACTTTGCTGTGA	74 pb
*FN1*	TCAGCTTCCTGGCACTTCTG	TCTTGTCCTACATTCGGCGG	147 pb
*COL1A1*	GCTCTTGCAACATCTCCCCT	CCTTCCTGACTCTCCTCCGA	88 pb
*VCAN*	AGTGTGTGCACGGAATGGAA	GTGGCTCTGGACACAACAGA	162 pb

### Tissue Staining and Image Processing

Fixed tissue samples were embedded in paraffin and cut into 4 µm thick sections, which were stained with hematoxylin and eosin for morphological analysis and with Masson's trichrome to assess the extent of tissue fibrosis.

The images were scanned (3DHISTEC Ltd., Budapest, Hungary) and fibrosis was quantified using ImageJ software, as described by Chen et al. [27]. Briefly, all images were acquired under the same light conditions, without automatic exposure and white balance, and digitalized with 24-bit and 640 × 480 pixel resolution. Digital images were processed by color deconvolution and the proportion of blue color, which corresponds to collagen fibers, was quantified from the selected areas of interest [27].

### Statistical Analysis

The results were analyzed by the D'Agostino-Pearson test to determine normal data distributions. Continuous variables were summarized as mean ± standard deviation or medians (interquartile ranges) and categorical variables were expressed as frequency (percentage). Differences between groups were assessed using the Kruskal–Wallis analysis of variance followed by Dunn’s test. All analyses were performed using GraphPad Prism version 6.0.

## Results

As shown in Table 1, the three groups were comparable in terms of age at the time of hysterectomy, age of menarche, parity, blood pressure, body weight and body mass index. Moreover, the groups were similar in clinical characteristics such as uterine volume, menstrual cycle length, hemoglobin levels and painful symptoms (Table 1).

### Self-reported Skin Color, Fibrosis Extent and Expression of Fibrosis Markers

**Fig. 1:**
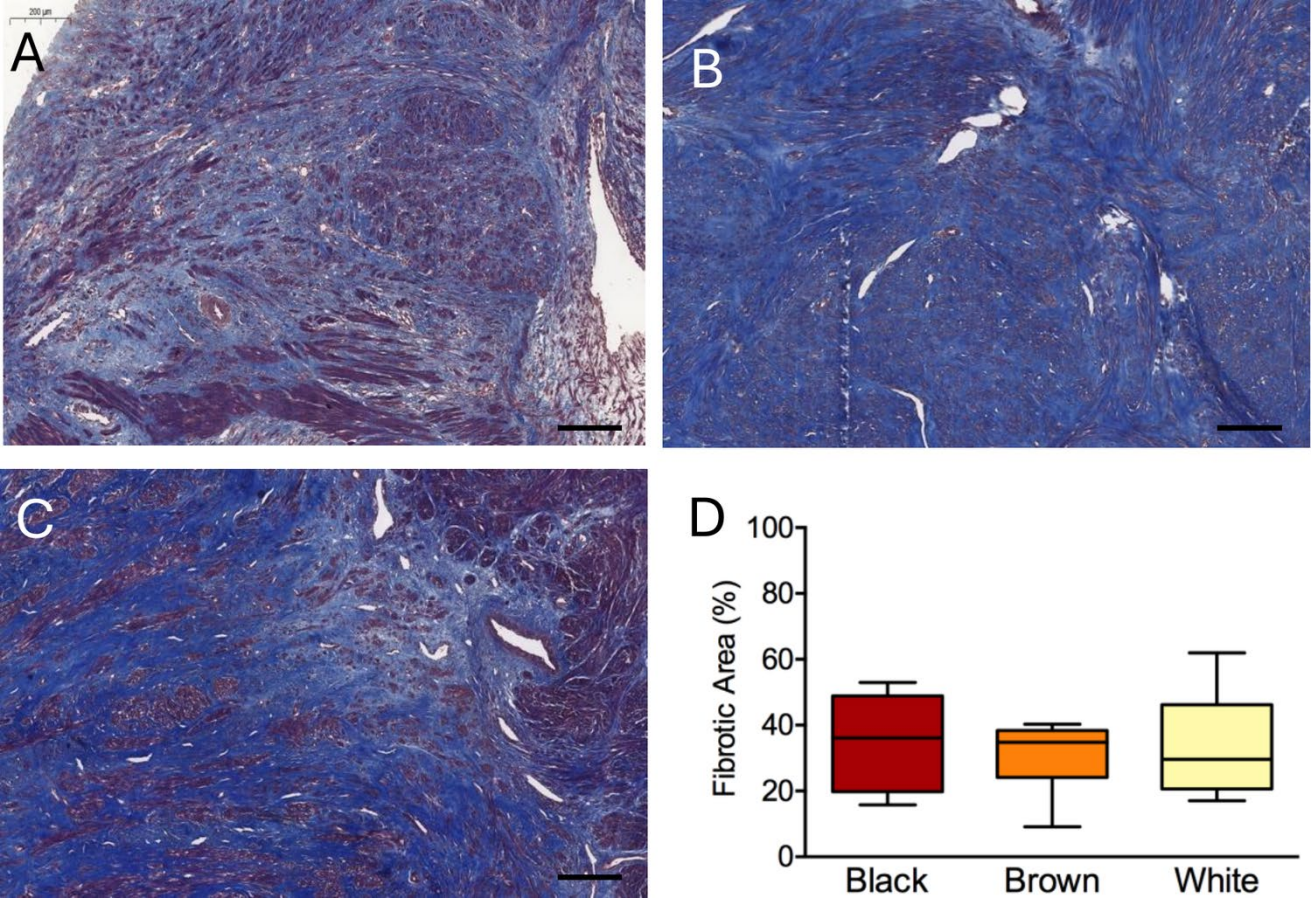
Extent of fibrosis in uterine leiomyoma tissue of Black (**A**), Brown (**B**) and White (**C**) women. The proportion of fibrotic area (**D**) is shown in the boxplots as group medians and interquartile ranges. Scale bar: 200 µm

As shown in Fig. 1, the proportion of fibrotic tissue in the ULM histological sections was similar in the three groups.

**Fig. 2:**
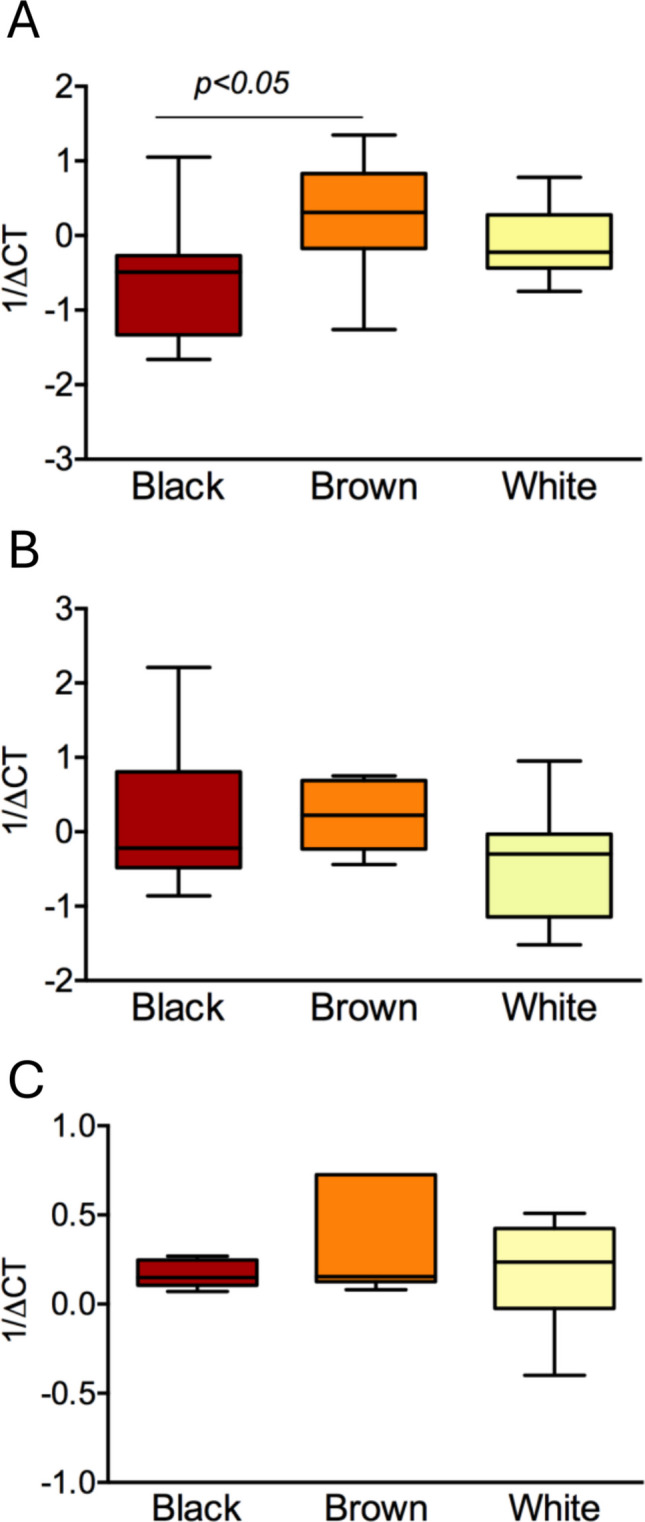
Relative mRNA expression of fibrosis-related genes *COL1A1* (**A**), *FN1* (**B**) and *VCAN* (**C**) in uterine leiomyoma tissue according to self-reported skin color/race

Black women presented a significantly lower expression (*p* < 0.05) of type I collagen mRNA compared to the Brown group. We observed no significant association between the expression of fibronectin or versican mRNA in ULM tissue and self-declared color (Fig. 2).

### Self-reported Color and Genetic Ancestry

**Fig. 3:**
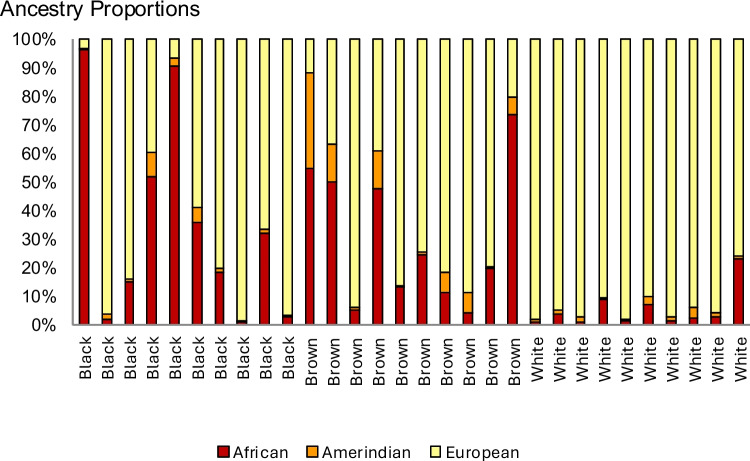
Standard estimates of patient ancestry from genotyping data

Self-reported Black color was not associated with a predominance of African genetic ancestry (Fig. 3). As expected, all participants in the White group had at least 75% European ancestry, while Brown color was associated with a more variable pattern of ancestry-related gene polymorphisms, including the highest proportion of Amerindian ancestry among the groups.

### Genetic Ancestry and Expression of Fibrosis Markers

**Fig. 4:**
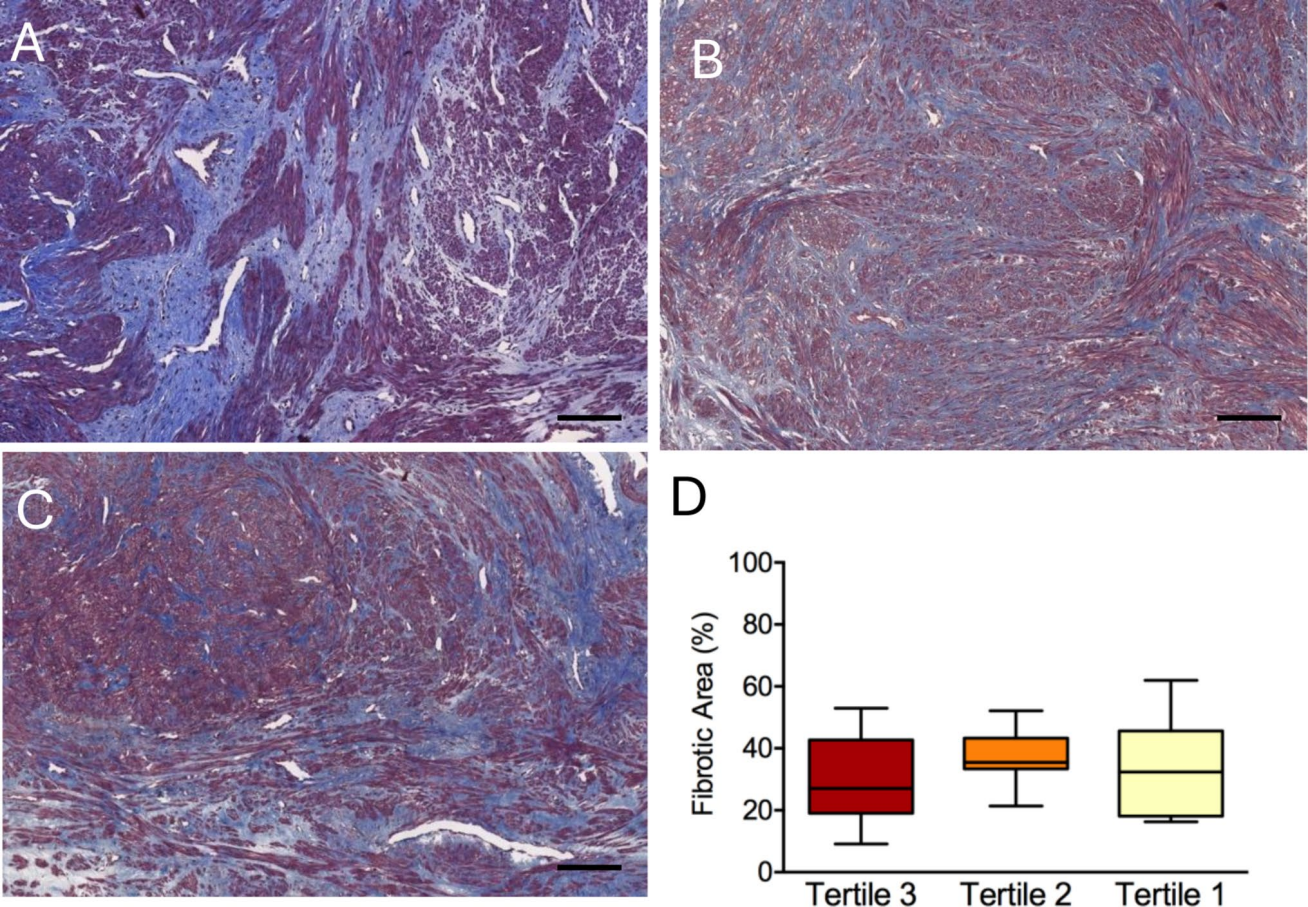
Extent of fibrosis in uterine leiomyoma tissue of women in the top (**A**), middle (**B**) and bottom (**C**) tertiles of African ancestry. The proportion of fibrotic area (**D**) is shown in the boxplots as group medians and interquartile ranges. Scale bar: 200 µm

**Fig. 5:**
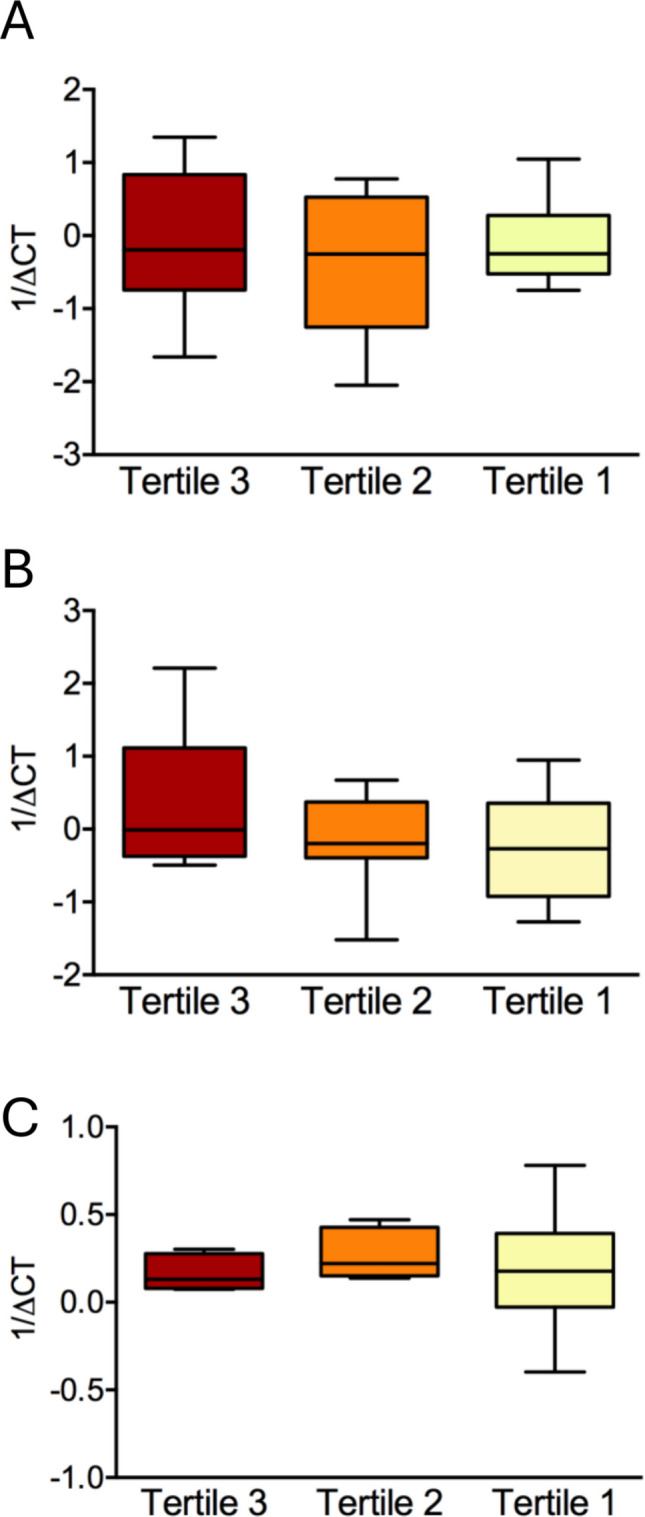
Relative mRNA expression of fibrosis-related genes *COL1A1* (**A**), *FN1* (**B**) and *VCAN* (**C**) in uterine leiomyoma tissue according to tertiles of African ancestry

When the study participants were reclassified into tertiles by the proportion of African ancestry in their genotype profiles, we observed a similar extent of fibrosis in all groups (Fig. 4). Likewise, the expression levels of type 1 collagen, fibronectin and versican did not show any meaningful difference according to the degree of African ancestry (Fig. 5).

## Discussion

The present study investigated the fibrosis extent and the expression of pro-fibrotic genes *COL1A1*, *FN1* and *VCAN* in ULM tissue of Brazilian women from different racial groups. As a result, the mRNA levels of type 1 collagen were lower in Black than in Brown group while fibronectin and versican expression did not change according to self-reported skin color/race. There was no correlation between fibrosis extent or pro-fibrotic gene expression and the proportion of European, African or Amerindian genomic ancestry of the participants.

Baterman et al. [21] demonstrated using shear wave ultrasonography that Black women had firmer ULMs than White patients, even when tumors were similar in size. Furthermore, Black women harbor *MED12* pathogenic variants, the most prevalent genetic alteration associated with sporadic ULMs and related to increased fibrosis, more frequently than White women. Bairiane et al. [16] evaluated the expression of pro-fibrotic genes (type I collagen and fibronectin) in the myometrium of uteri with or without ULMs and observed an increase in the levels of both markers in ULM-associated myometrium, but this difference was only seen among Black women. These results together with previously accumulated evidence indicate that the racial disparities observed in ULMs may be attributed, at least in part, to exacerbated production of ECM in the myometrium of Black women, even before the appearance of the tumors [16].

To the best of our knowledge, no previous study made direct comparisons between ethnic or racial groups for the severity of fibrosis in ULMs. Based on the known racial differences in tumor progression and symptoms [1, 18] and considering the central role of fibrotic tissue in ULM pathophysiology [10], we hypothesized that the tumor would have a greater proportion of fibrosis in Black women. However, this hypothesis was not supported by the present findings. In the present study, a possible explanation for the similarity in fibrosis extent between racial groups may be because all ULM samples were obtained from symptomatic patients with indication for hysterectomy, i.e., they presented an advanced stage of the disease. In fact, all three groups had similar uterine volume, menstrual cycle length, hemoglobin levels and painful symptoms. Nevertheless, it is still possible that such racial differences in ECM deposition and fibrosis exist in early stages of ULM growth and even in the myometrium before tumoral transformation [16].

The strengths of this study include the prospective design with strict selection criteria and the assessment of genetic ancestry to complement the investigation. A further strength is the demographic profile of the Brazilian population, with diverse and mixed racial and ethnic groups [28], which adds to the existing knowledge on ULM pathophysiology obtained mostly from more homogeneous racial groups. A study limitation is that samples were collected at a time of symptomatic and advanced disease. In addition, we assessed only three pro-fibrotic genes, although several other genes related to fibrosis have already been documented [10, 11, 16].

In conclusion, the present findings suggest that differences in the amount of fibrosis or the expression of pro-fibrotic genes by the time of hysterectomy are unlikely to explain racial disparities in the burden of ULMs among Brazilian women. Further investigation of the molecular mechanisms underlying the particularities of ULM in Black women is of paramount importance to subside more precise and tailored interventions, especially for patients who do not respond to current medical treatments and end up in hysterectomy.

## Data Availability

The datasets generated during the current study are available from the corresponding author on request.
